# Carbon–carbon bond formation using aromatics from biomass

**DOI:** 10.1039/d4cc05664g

**Published:** 2024-11-27

**Authors:** Petter Dunås, Andrew J. Paterson, Simon E. Lewis, Nina Kann

**Affiliations:** a Department of Chemistry and Chemical Engineering, Chalmers University of Technology SE-41296 Göteborg Sweden kann@chalmers.se; b Department of Chemistry, University of Bath Convocation Avenue Bath BA2 7AY UK S.E.Lewis@bath.ac.uk; c Institute of Sustainability and Climate Change, University of Bath Bath BA2 7AY UK

## Abstract

The transition to a circular economy requires that we adapt currently used chemical processes to the structurally diverse and often highly oxygenated precursors that are accessible from biomass. In this review, we highlight different examples of carbon–carbon bond formation using aromatics derived from bio-based sources, reported during 2015–2024. Examples of sustainable biomass building blocks include heterocycles such as furfural and hydroxymethylfurfural, obtained from carbohydrates, as well as lignin-based aromatics such as vanillin and eugenol. These have subsequently been applied in a variety of different types of carbon–carbon bond formation, including more classical methods such as aldol condensation and Morita–Baylis–Hillman reactions, but also employing transition metal catalysis, electrochemistry or photochemistry to create new C–C bonds.

## Abstract 2

This highlight provides an overview of methods for carbon–carbon bond formation using sustainable aromatics derived from biomass.

## Graphical abstract

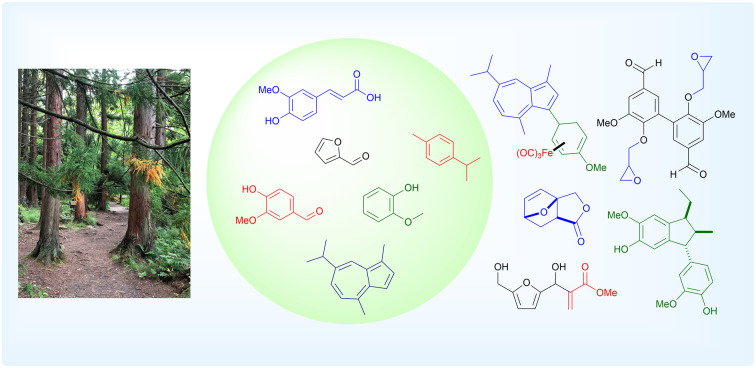

## 1. Introduction

Precursors for organic synthesis and its applications are currently to a large extent derived from petroleum. In the ongoing shift to a more sustainable circular economy, we need to continue investigating renewable sources, such as lignocellulosic biomass, to access the building blocks that are necessary for fuel, materials and pharmaceuticals.^1,2^ A challenge in this endeavour is that bio-based precursors generally contain more oxygen atoms than molecules derived from petroleum, making it difficult to directly replace chemicals currently used in industry.^3^ Some adaptions to the processes we use for transforming these sustainable building blocks into other chemical structures are thus required. This can be addressed *via* deoxygenation, *i.e.* chemical removal of oxygen-containing functional groups, to make the chemical structures more similar to those derived from petroleum and currently used in industry, and this is a highly active research area.^4^ Another strategy is instead to exploit the functional groups already present in molecules derived from biomass, such as alcohols and aldehydes found in carbohydrates and lignin fragments, for further chemical transformations. The higher oxygen content in biomass-derived aromatics also renders them well suited for certain transformations that require electron-rich structures, such as oxidative coupling and reactions that exploit the arenes as nucleophiles. In this highlight, we will look more closely at aromatic building blocks derived from biomass sources and how they can be applied in carbon–carbon bond formation reactions.

**Fig. 1:**
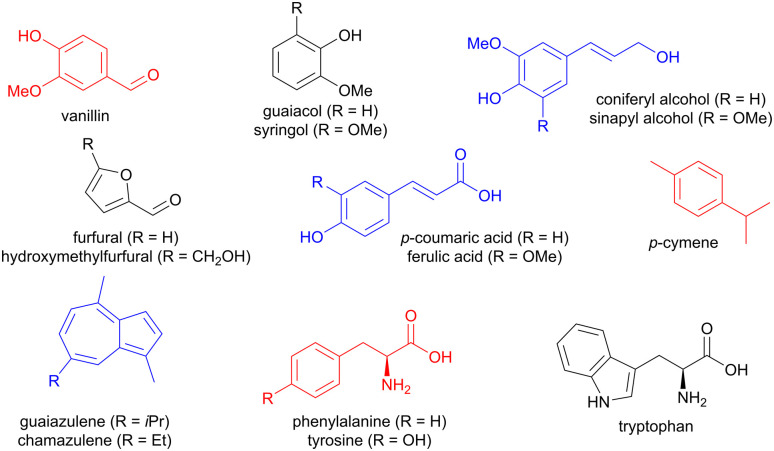
Examples of aromatic molecules available from biomass.

Lignin is a rich source of small aromatic molecules, accessible *via* depolymerization reactions involving C–O or C–C cleavage,^5–8^ and affording for instance vanillin, guaiacol, syringol, as well as sinapyl and coniferyl alcohols, but also simpler structures like benzaldehyde (Fig. 1).^9^ Carboxylic acids such as ferulic acid and *p*-coumaric acids can also be obtained.^9^ Arenes with other ring sizes, such as the brightly coloured chamazulene and guaiazulene, are accessible from plant material such as chamomile and yarrow.^10^ In addition, carbohydrates such as fructose and xylose can be chemically transformed into 5-membered furan derivatives, mainly furfural and hydroxymethylfurfural.^11^ Chitin, a biopolymer found in the exoskeleton of insects, is also a source of furans.^9^ Food waste provides aromatic amino acids, such as phenylalanine, tyrosine, and tryptophan,^12^ while terpenes can be chemically modified to access aromatics such as *p*-cymene from limonene or α-pinene.^9^

This highlight does not strive to provide an exhaustive review of the use of bio-based aromatics in carbon–carbon bond forming reactions, but rather to show examples of precursors that have been employed and chemical transformations that can be applied in this context. Reactions include not only classical C–C bond forming reactions, such as aldol condensations and Diels–Alder cycloadditions, but also methods catalyzed or mediated by transition metal complexes, or that use electrochemical or photochemical transformations.

## 2. Diels–Alder reactions

**Scheme 1:**
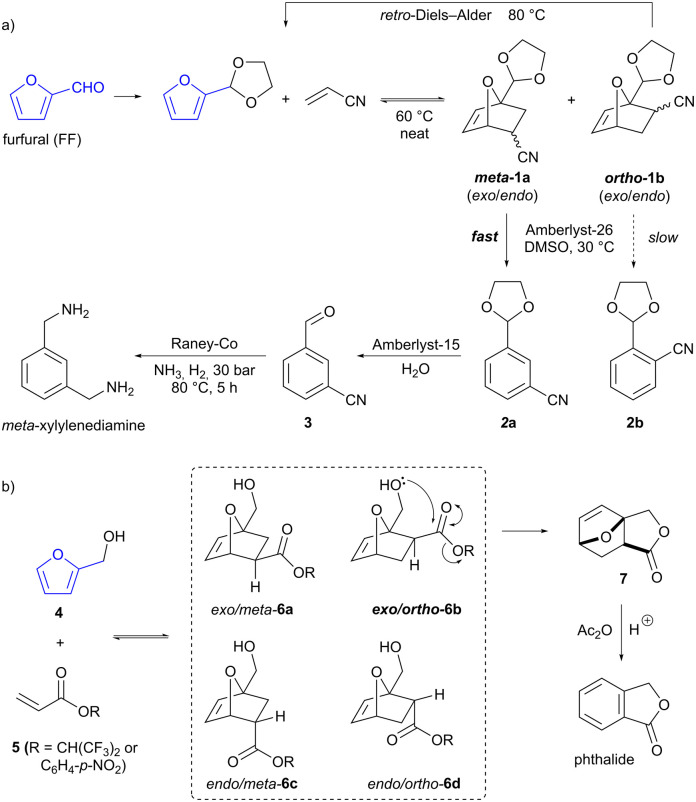
Furanic Diels–Alder reactions for the production of (a) *meta*-xylylenediamine and (b) phthalide.

A major route to aromatics from biomass is by the acid-catalysed dehydration of carbohydrates,^11^ which allows access to furan-containing products such as furfural (FF) and hydroxymethylfurfural (HMF). Production of FF from feedstocks such as shelled corncobs is an established industrial process operated at a scale of ≈450 kT per annum.^13^ Furans are able to serve as dienes in Diels–Alder reactions, forming two C–C bonds in a single step. The resultant oxabicyclo[2.2.1] adducts can then be converted into various final products, including benzenoid aromatics by dehydration.^9^ A potential issue with such furan Diels–Alder reactions is that multiple regio- and stereoisomeric products may be formed (as is the case for any cycloaddition between non-symmetrical substrates). However, Diels–Alder reactions of furan are also often reversible (unlike those of most other dienes), due to the regeneration of aromaticity by the retro-cycloaddition process. This provides opportunities to exploit differences in the reactivity of the various product isomers, in order to influence selectivity. Thus, Wischert, Jérôme and co-workers have developed a regioselective synthesis of the commodity chemical *meta*-xylylenediamine by the Diels–Alder reaction of acetal-protected FF and acrylonitrile (Scheme 1a).^14^ The reaction forms both the *meta* (1a) and (undesired) *ortho* (1b) regioisomers of the cycloaddition product in roughly equal proportions (and each of these is a mixture of the respective *exo* and *endo* stereoisomers). However, when the product mixture is exposed to base (basic resin Amberlyst-26 or alkoxide bases) in DMSO, ring-opening and dehydration to form 2a and 2b occurs significantly faster for the *meta* isomers than the *ortho*. This difference is ascribed to greater acidity of the nitrile α-proton in the *meta* isomers. Quenching the reaction at 50% conversion gave the desired *meta*-substituted benzonitrile 2a as a mixture with the unreacted *ortho*-cycloadducts. These *ortho*-cycloadducts could be recycled by increasing the temperature to effect the retro-Diels–Alder reaction. The desired *meta* product underwent acetal hydrolysis (with acidic resin Amberlyst-15) to give aldehyde 3, followed by imine formation and reduction over RANEY® cobalt to give the final product *meta*-xylylenediamine.

Bruijnincx and co-workers have reported the synthesis of another commodity chemical, phthalide, *via* the Diels–Alder reaction of furfuryl alcohol (4, produced by reduction of bioderived FF) and an acrylate ester (5, Scheme 1b).^15^ Here again, the cycloaddition exhibits little selectivity, and all four possible regio- and stereoisomers of the cycloaddition product 6 are formed in equilibrium with the starting materials. The hydroxymethyl sidechain in the cycloadducts can potentially act as a tethered nucleophile, but in only one of the four isomers (the *exo*/*ortho* isomer 6b) can it adopt the required conformation for attack on the ester carbonyl. Therefore, only this isomer undergoes addition to the carbonyl to give the tricyclic lactone product 7. Crucially, this step is irreversible and moreover, the lactone is seemingly not able to undergo a retro-Diels–Alder reaction itself under the reaction conditions. Accordingly, dynamic kinetic trapping ensures that all cycloadduct isomers equilibrate to the *exo*/*ortho* isomer 6b and are ultimately converted to the tricyclic lactone 7. Subsequent treatment of this with acetic anhydride in the presence of a Brønsted acid catalyst effects the dehydration/aromatization to give phthalide in 84% overall yield.

## 3. Pictet–Spengler cyclization

**Scheme 2:**
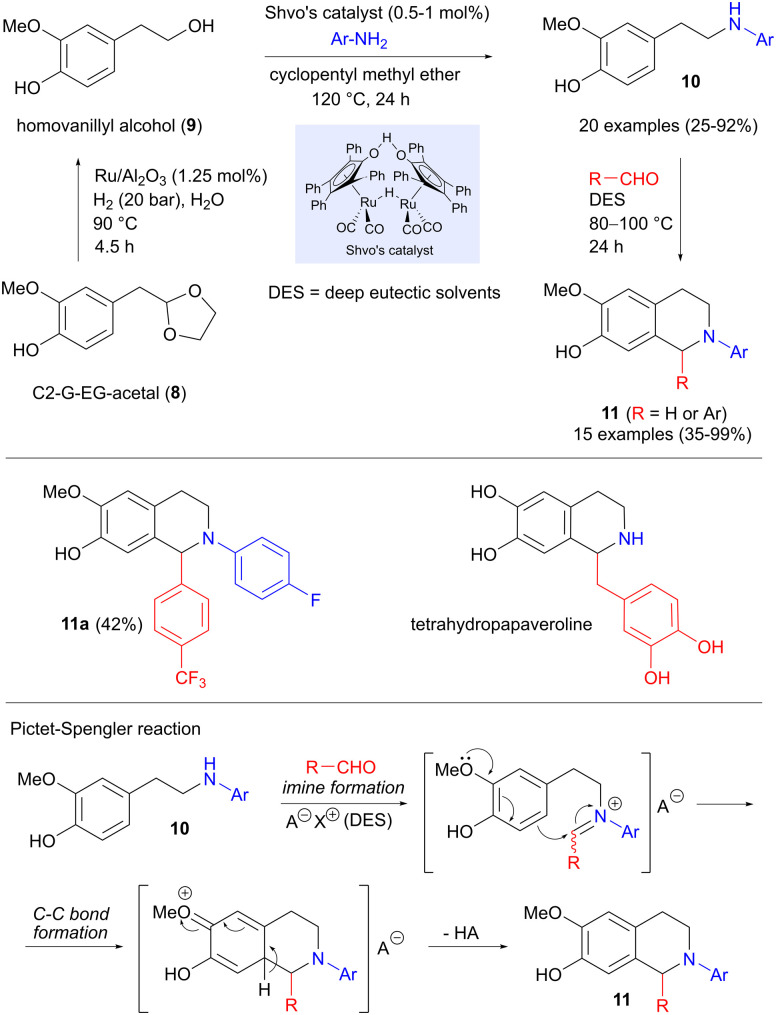
Synthesis of tetrahydroisoquinolines from a lignin platform chemical *via* hydrogen borrowing and Pictet–Spengler reactions.

While much of the research on replacing petroleum-derived precursors with biomass-based building blocks has focused on fuel and new materials, an equally important but less explored area is the production of pharmaceuticals. In this context, Hirsch and Barta have investigated whether a lignin-derived platform molecule can be employed in the synthesis of active pharmaceutical ingredients (APIs).^16^ The precursor used here is C2-G-EG-acetal (8, Scheme 2), which can be obtained in high yield from softwood lignin by acidic hydrolysis, followed by trapping of the intermediate aldehyde as an acetal.^17^ Concomitant deprotection and reductive hydrogenation of 8 to homovanillyl alcohol 9 was effected in excellent yield under ruthenium catalysis, using water as the solvent. Continuing with ruthenium catalysis, but now using Shvo's catalyst in a hydrogen borrowing reaction (see also Section 5.4),^18,19^ the alcohol was coupled with anilines to form amines 10, as shown in Scheme 2. The carbon–carbon bond formation takes place in the final step, which involves a Pictet–Spengler reaction between 10 and various aldehydes (formaldehyde or benzaldehydes), producing 15 different tetrahydroisoquinolines 11 in 35–99% yield. An important feature of this reaction is the use of deep eutectic solvents based on natural products (choline and oxalic acid), whose acidity could be fine-tuned to optimize the yields. Of particular interest here was product 11a, which displayed excellent antibacterial activity against *Streptococcus pneumoniae*, as well as interesting cytotoxic behaviour. A similar strategy was also used to prepare the natural product tetrahydropapaveroline, albeit using a different amination procedure.

**Scheme 3:**
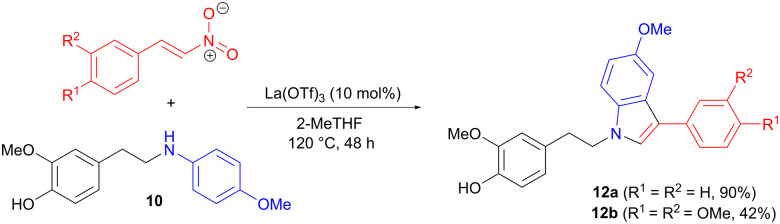
Synthesis of indole derivatives *via* amine 1,4-addition to a nitrostyrene, followed by aromatization.

Two of the intermediate amines 10 were also applied in 1,4 addition to *trans*-β-nitrostyrenes (Scheme 3), involving modifications of a reported protocol,^20^ in order to tailor the reaction conditions to lignin-based substrates. The aromatic ring of the aniline acts as the nucleophile in a 1,4-addition to the nitrostyrene, forming the carbon–carbon bond. Indoles 12 are then produced in moderate to good yields in a final cyclization-aromatization step.

## 4. Reactions involving enolates, including organocatalytic methods

**Scheme 4:**
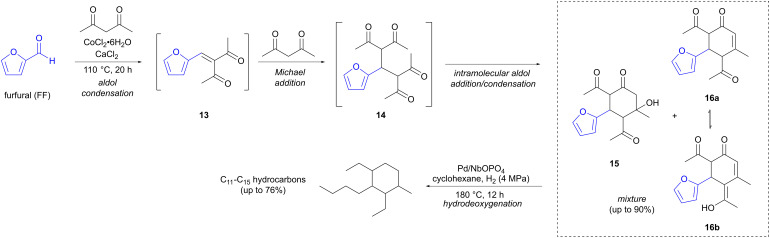
Synthesis of branched hydrocarbons, suitable for jet fuel applications, *via* sequential aldol reactions involving furfural.

Reactions employing enolates as nucleophiles, such as aldol and Claisen condensations, are classical methods for C–C bond formation. Pioneering work on aldol reactions involving FF and HMF as precursors has been reported by Dumesic and Huber.^21–24^ Several of the more recent examples involving enolates focus on finding biomass-derived alternatives for fuel for transportation. Jet fuel needs a higher proportion of branched hydrocarbons compared to fuel for cars, in order to function at lower temperatures. Wang and co-workers have developed an elegant two-step method, where furfural (FF) undergoes a Robinson annulation, followed by hydrodeoxygenation, to provide cyclic branched C_11_–C_15_ hydrocarbons (Scheme 4).^25^ Reaction of FF with 2,4-pentadione, which can be sourced from lignocellulose, in the presence of a Lewis acid (CoCl_2_·6H_2_O), triggers a reaction sequence that initiates with an aldol condensation between the two reactants, forming adduct 13, which is followed by a Michael addition of a second equivalent of the diketone to form intermediate 14. A cyclative intramolecular aldol reaction then ensues, forming a mixture of aldol addition (15) and condensation products (tautomers 16a and 16b), in up to 90% yield of C_15_ oxygenates for the one-pot reaction. It is this step that provides the crucial cycloalkane skeleton, needed to increase the density and to decrease the freezing point of the fuel. The product mixture is then converted to pure hydrocarbons in a second reaction step, where hydrodeoxygenation takes place, effected by hydrogen gas (4 MPa) in the presence of a solid metal catalyst (Pd/NbOPO_4_). This provides a mixture of C_11_–C_15_ hydrocarbons in up to 76% yield, with the C_11_ and C_13_ hydrocarbons resulting from hydrocracking of the ethyl side chains. C_20_ hydrocarbons, could also be accessed by using a longer reaction time (36 h) and a higher temperature (140 °C) for the Robinson annulation step. The additional carbon atoms here arise from the reaction of aldol products 15 and 16 with a third equivalent of 2,4-pentadione in a further aldol reaction. The hydrocarbons formed *via* hydrodeoxygenation of these C_20_ aldol products could find potential applications as lubricants.

**Scheme 5:**
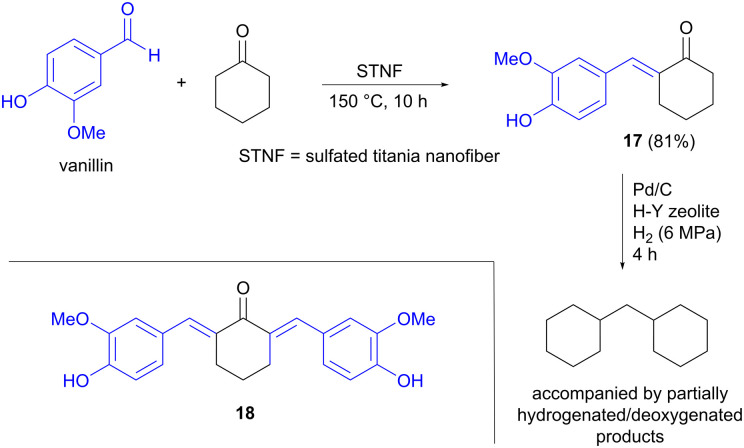
Jet fuel range polycycloalkanes prepared from vanillin.

Jet fuel-range polycycloalkanes can also be prepared from vanillin using an aldol condensation strategy, as described by Li, Wang and Li, in this case starting from two cyclic precursors.^26^ Vanillin is one of few aromatic building blocks that is produced on an industrial scale from lignin today, where the processes used can compete with petroleum-based routes.^9,27^ The other carbonyl compound used in the aldol reaction was cyclohexanone, which can be accessed *via* hydrogenation of lignin-derived phenols such as anisole.^28^ The aldol condensation was performed at 150 °C under solvent-free conditions, in the presence of a solid titanium-based catalyst, affording mainly the single aldol condensation product 17, accompanied by smaller amounts of the double aldol product 18 (Scheme 5). Several titanium catalysts were compared, but sulfated titania nanofiber (STNF) afforded both the highest conversion of vanillin as well as the highest yield of 17, showing similar activity to commercial acidic catalysts such as Amberlyst-15 resin. The higher acidity of STNF compared to the other Ti-based catalysts investigated was proposed to explain the better performance. Hydrodeoxygenation of 17 using Pd/C, in the presence of a H–Y zeolite, was also carried out to demonstrate the potential for converting the aldol products into non-oxygenated hydrocarbons.

**Scheme 6:**
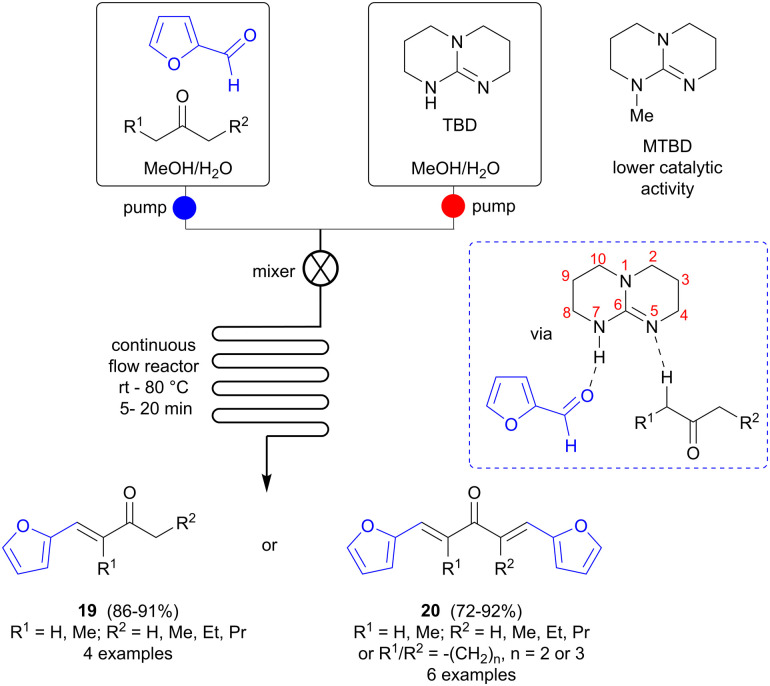
Aldol condensations of furfural (FF) using continuous flow methodology.

Continuous flow synthesis can offer advantages over batch reactions in terms of safety, efficiency, improved energy transfer and facile scale-up, and has more recently also been adapted to accommodate multistep reactions.^29^ Zhu and colleagues investigated if continuous microflow synthesis could be a versatile method for preparing bio-based fuel precursors from FF and various ketones (Scheme 6).^30^ A soluble catalyst is needed in this case and several amines and amino acids, *i.e.* 1,4-diazabicyclo[2.2.2]octane (DABCO), *i*PrNEt_2_, l-proline and l-tryptophan, were screened in the reaction of FF with 3-pentanone (batch mode), but failed to catalyze the reaction. Bicyclic amidines and guanidines, such as 1,8-diazabicyclo [5.4.0]undec-7-ene (DBU) and 1,5,7-triazabicyclo[4.4.0]dec-5-ene (TBD) were found to be more successful, however, and TBD was selected for further studies. The ratio of single/double aldol condensation product could be controlled simply by changing the ratio of FF to ketone, selectively affording either 19 or 20 in good yields. To avoid precipitation during the flow process, which might cause blocking and malfunction of the flow system, a solvent that can dissolve all reaction components (substrates, reagents, intermediates and products) is needed and solvent screening showed that a 1 : 1 mixture of methanol and water fulfilled this role. Side reactions could be suppressed and the reaction time shortened to 5–20 minutes using flow synthesis, in comparison to several hours for batch mode. The catalyst is proposed to have a synergistic effect, not only acting as a base in the ketone deprotonation to form the enolate, but also participating by hydrogen bonding to the FF oxygen *via* the H-N7 functionality. To verify this hypothesis, a similar bicyclic guanidine, 7-methyl-1,5,7-triazabicyclo[4.4.0]dec-5-ene (MTBD), where the N7 hydrogen has been replaced by a methyl group, was also tested, but was found to be a less active catalyst than TBD, with a substantially lower reaction rate, thus supporting this theory.

**Scheme 7:**
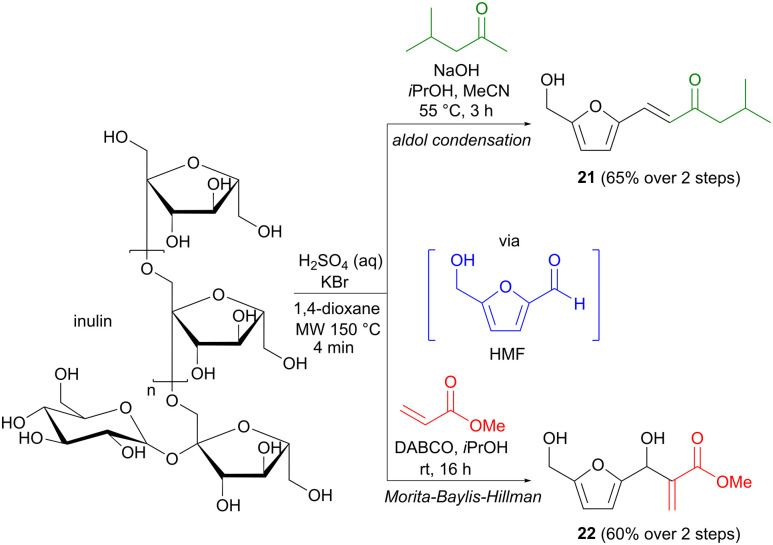
One-pot transformations of inulin to aldol/Morita–Baylis–Hillman products *via* hydroxymethylfurfural (HMF).

Apart from the more classic aldol methodology, the Morita–Baylis–Hillman reaction provides a different means of enolate addition to a carbonyl group.^31^ The enolate is in this case formed *via* the conjugate addition of a nucleophilic organocatalyst to an activated alkene, where the latter is generally in the form of an α,β-unsaturated aldehyde, ketone or ester. As mentioned, hydroxymethylfurfural (HMF) is a biomass platform chemical, which despite its aromatic structure, has a carbohydrate origin.^32^ While most carbon–carbon bond formations involving HMF employ commercially produced material, Repo and colleagues have taken this one step further in developing one-pot transformations where HMF is formed *in situ* and used directly in a second reaction step.^33^ On industrial scale, HMF is generally prepared from fructose,^32^ but Repo found that the more complex carbohydrate inulin could also be used as a precursor in this context. Under acidic conditions, in the presence of KBr, inulin was transformed to HMF in only a few minutes using microwave heating with 1,4-dioxane as the solvent (Scheme 7). The role of KBr is to facilitate the initial dehydration process *via* the formation of an intermediate 2-bromofructofuranose, which is subsequently converted to HMF *via* the elimination of two equivalents of water. Additional reagents were then added directly to the microwave vial to perform the second step, involving either an aldol reaction with methyl isobutyl ketone, affording 21 in 65% yield, or a Morita–Baylis–Hillman transformation with methyl acrylate, affording 60% of adduct 22. Both yields are calculated for the two-step sequence and conventional oil-bath heating was used for the aldol reaction step. Several other one-pot transformations were also demonstrated (oxidation, reduction, Cannizzaro reaction), but these do not involve carbon–carbon bond formation. This direct and practical methodology provides an important contribution to the toolbox of transformations involving biomass platform chemicals.

## 5. Reactions catalyzed or mediated by transition metals

In addition to using renewable feedstocks, employing catalytic methods when possible, rather than utilizing stoichiometric reagents to effect a chemical transformation, is one of the 12 Principles of Green Chemistry outlined by Anastas and Warner in 1998.^34^ The use of organocatalysis for Morita–Baylis–Hillman reactions has already been mentioned, but transition metal catalysis provides another opportunity for conducting efficient transformations without excessive waste from spent reagents. Two examples of stoichiometric reactions will also be included here, but using earth-abundant iron as the metallic component.

### 5.1 Copper

**Scheme 8:**
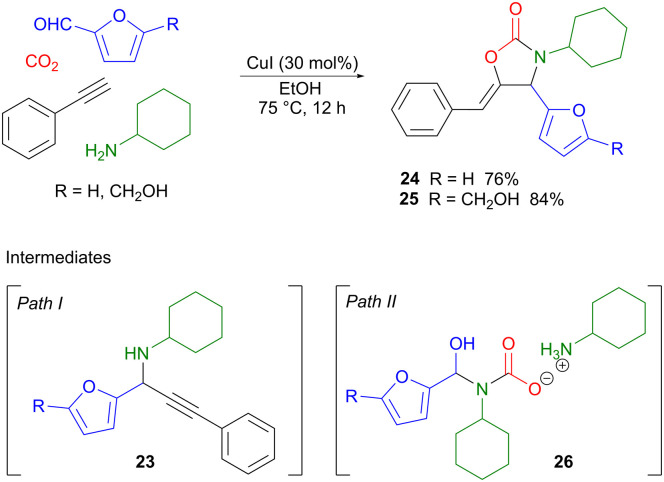
A Cu(i)-catalyzed four-component reaction employing renewable furfural (FF) and hydroxymethylfurfural (HMF).

A copper(i)-catalyzed four-component reaction was used by Xu to couple two different classes of renewable building blocks, *i.e.* carbon dioxide and bio-based aldehydes such as furfural or HMF with terminal aromatic alkynes and primary aliphatic amines, affording 1,3-oxazolidin-2-ones as the products.^35^ Oxazolidinones are useful intermediates in organic synthesis, in particular as chiral auxiliaries,^36^ but are also important pharmaceutical components, for example in broad-spectrum antibiotics such as linezolid.^37^ Two different reaction pathways were proposed for the four-component reaction (Scheme 8). Path I involves the amine-assisted formation of a copper acetylide which subsequently performs a nucleophilic attack on the imine formed from the amine and the aldehyde, forming intermediate 23. This is also the product isolated if the reaction is carried out in the absence of CO_2_. Amine carboxylation, followed by Cu(i)-promoted cyclization, then affords the target oxazolidinone 24 or 25. Path II provides an alternative scenario, where the amine first reacts with CO_2_ to form a carbamic acid ammonium salt which subsequently reacts with the aldehyde, producing intermediate 26. Displacement of the hydroxyl group by the copper acetylide, followed by cyclization, then completes the reaction. Control experiments showed that both reaction pathways were feasible and could potentially occur in parallel in a competitive fashion depending on the conditions used. Optimization of the reaction conditions showed that when using CuI as the catalyst, in conjunction with a sustainable protic solvent such as ethanol, heating at 75 °C for 12 hours, products 24 and 25 were produced in 76% and 84% yield, respectively. The use of heteroaromatics on the alkyne gave low product yields, while variation of the amine component was in general well tolerated. Given that bio-based amines are available *via* catalytic amination of renewable alcohols,^38,39^ and that lignin-derived aldehydes can be converted into terminal alkynes,^40^ there is the potential for extending this methodology so that also these two components derive from biomass.

### 5.2 Iron

**Scheme 9:**
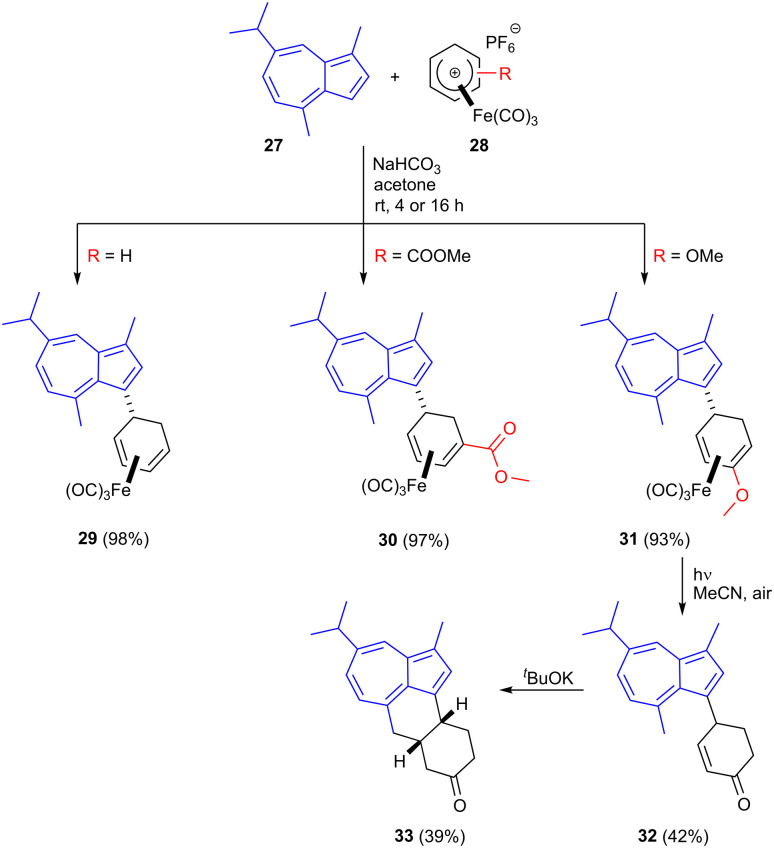
Derivatization of guaiazulene using cationic iron carbonyl dienyl complexes.

Guaiazulene 27 is a bioderived sesquiterpene aromatic compound used as a cosmetic additive and its derivative sodium guaiazulene-3-sulfonate is a licensed anti-inflammatory agent.^41^ It is produced industrially by the dehydrogenation/dehydration of essential oils from species in genera including *Eucalyptus* and *Matricaria* (chamomile).^42–44^ Azulenes readily undergo S_E_Ar reactions at their 1- and 3-positions and the present authors have shown that cationic η^5^-iron carbonyl cyclohexadienyl complexes 28 readily react at the guaiazulene 3-position resulting in C–C bond formation in products such as 29–31 in high yield using mild reaction conditions (Scheme 9).^45^ The formed iron carbonyl complexes could be demetallated under UV radiation, liberating the free dienes. For the methoxy substituted product 31, demetallation was followed by enol ether cleavage to give the corresponding enone 32. Treatment of this enone with base led to deprotonation of the guaiazulene C4 methyl group, followed by Michael addition; this second C–C bond formation gave tetracyclic product 33.

**Scheme 10:**
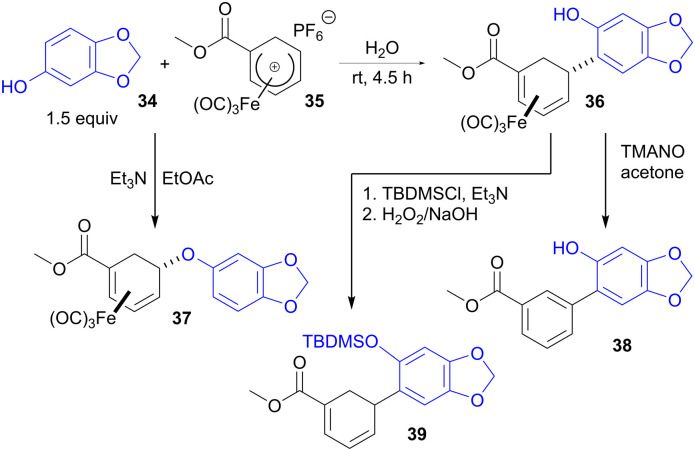
Derivatization of bio-based phenols with C–C or C–O selectivity using iron carbonyl dienyl complexes.

Bioderived phenols have also been employed as ambident nucleophiles capable of forming either C–C or C–O bonds to cationic η^5^-iron carbonyl cyclohexadienyl complexes, depending on the reaction conditions. For example, we have shown that naturally occurring sesamol (34) reacts with iron complex 35 (obtained from bio-derivable furan *via* a cycloaddition)^46^ to form C-linked adduct 36 in water or ethanol (Scheme 10).^47^ However, the same two reactants give isomeric O-linked product 37 when the reaction is performed in the presence of base and in an aprotic solvent such as EtOAc. Complex 36 can be demetallated with trimethylamine-*N*-oxide (TMANO) with concomitant dehydrogenation to biaryl 38. Alternatively, hydroxyl protection followed by demetallation with hydrogen peroxide disengages the η^4^ ligand without aromatisation to give diene 39.

### 5.3 Molybdenum

**Scheme 11:**
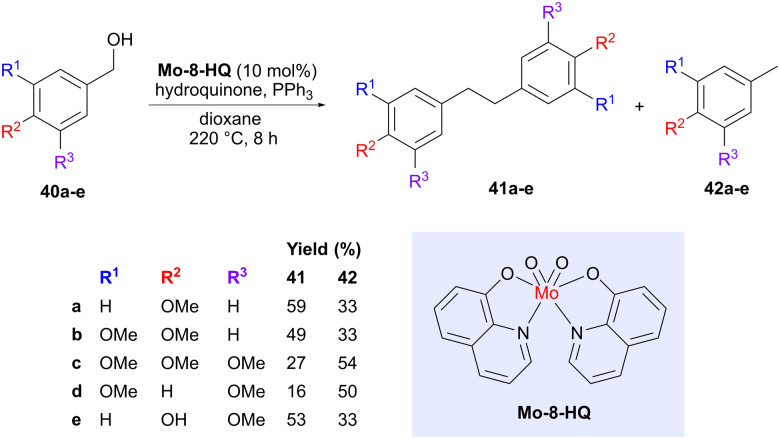
Molybdenum-catalyzed reductive coupling of bio-based benzylic alcohols.

As mentioned in the introduction, deoxygenation or defunctionalization of lignin-derived building blocks is one strategy to access building blocks that more resemble the hydrocarbons we currently obtain from fossil sources. However, such deoxygenation processes can also be exploited to simultaneously accomplish a coupling reaction. One such example was described by Lu and co-workers, who used a molybdenum catalyst (Mo-8-HQ) to convert various benzylic alcohols (40) obtained from lignin into deoxygenated bibenzyl products 41 (Scheme 11).^48^ The motivation for choosing this particular catalyst is that nitrogen donor ligands can enhance the electron density on molybdenum, which may be beneficial for the deoxygenation process.^49^ A catalyst screening also showed that Mo-8-HQ afforded a high conversion and good selectivity for the coupled bibenzyl product 41, compared to oxidation or reduction of the benzylic alcohol, which lead to the two main side products. The exact position of the –OMe groups was shown to affect the product distribution between the bibenzyl products 41, formed *via* deoxygenation with concomitant coupling, and the tolyl derivatives 42, formed *via* deoxygenation without coupling. Vanillyl alcohol (40e) was also evaluated, showing a preference for the bibenzyl product. The reaction was proposed to proceed *via* benzyl radical intermediates. Bibenzyls are important components in thermosetting plastics and can potentially act as replacements for the reprotoxic monomer bisphenol A.^50^

### 5.4 Ruthenium

**Scheme 12:**
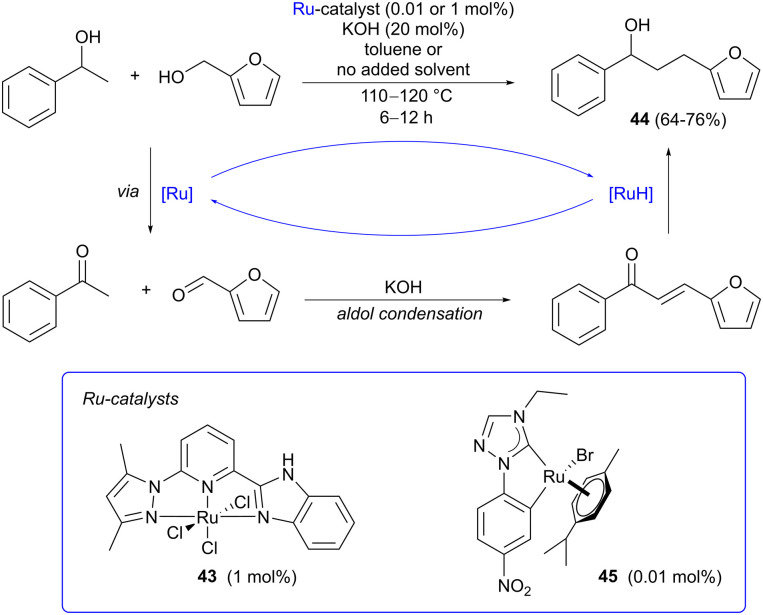
Ketone alkylation *via* ruthenium-catalyzed hydrogen borrowing using furfuryl alcohol as the alkylating agent.

Hydrogen borrowing involves the atom economic transition metal-catalyzed coupling of an alcohol with a nucleophile (Scheme 12),^18,19,51^ and has been extensively applied in the derivatizaton of bio-based alcohols,^52–54^ such as ethanol^55^ and glycerol.^56–59^ While most reports concern the amination of aliphatic alcohols, carbon–carbon bond formation *via* alkylation of enolate nucleophiles with alcohols is also possible and this approach has been applied in a few cases using furfuryl alcohol. Alkylation of 1-phenylethanol with furfuryl alcohol has been reported by both Yu^60^ and Rit^61^ with good results (Scheme 12). Yu employed pincer-type Ru(iii) catalyst 43 with NNN coordination, forming the target product 44 in 76% yield under solventless conditions. Rit instead applied Ru(ii)-NHC complex 45, prepared from commercially available [Ru(*p*-cymene)Cl_2_]_2_, affording 44 in a slightly lower yield (64%), using toluene as the solvent and a low catalyst loading (0.01 mol%). In both cases, potassium hydroxide was used as the base to generate the enolate nucleophile. Other aromatic bioalcohols such as vanillyl alcohol have been aminated under transition metal catalysis,^62^ but there are as yet no reports of *C*-alkylation *via* hydrogen borrowing using this particular substrate.

## 6. Electrochemistry

**Scheme 13:**
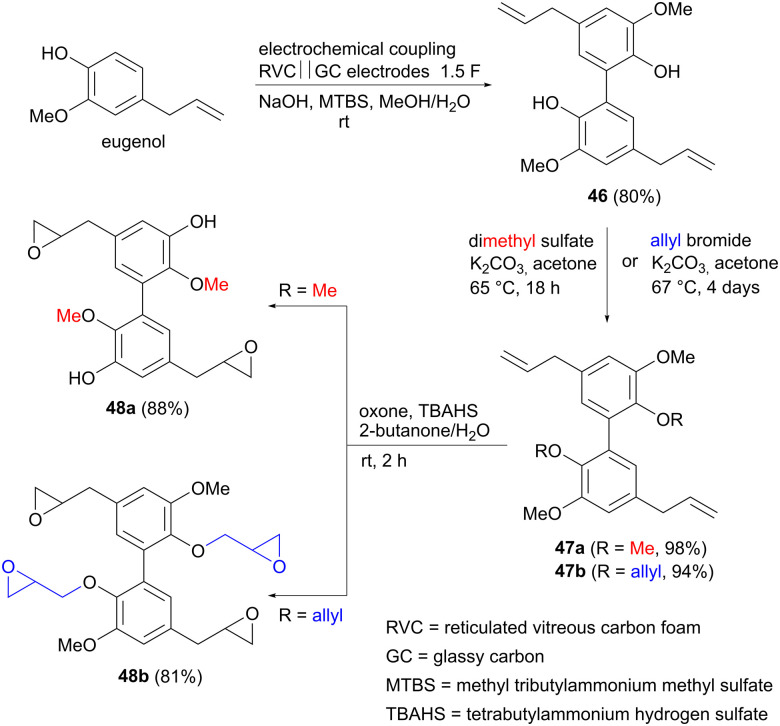
Electrochemical synthesis of biseugenol epoxide monomers for polymer applications.

Organic electrochemistry has seen a renaissance recently, with many new electrocatalytic reactions under development, giving access to functional group transformations that may be difficult to achieve using conventional organic synthetic methods.^63–65^ One such example is the oxidative coupling of phenols *via* a carbon–carbon bond, affording the corresponding dimerized products with high selectivity. Such coupling reactions were reported already in 2006, by Waldvogel and colleagues,^66^ and have since then been extended also to other functionalized aromatics,^67^ such as anilines^68^ and arylated pyridines.^69^ Three examples of electrocatalytic phenolic dimerization using renewable precursors will be mentioned here. Eugenol is found in clove oil, but can also be obtained *via* lignin depolymerization, and has been investigated as potential monomer for preparing bio-based polymers.^70^ In this context, Waldvogel and co-workers explored whether dimerized eugenol could be used as a replacement for reprotoxic bisphenol A in epoxy resins.^71^ Using reticulated vitreous carbon (RVC) anodes and glassy carbon (GC) cathodes, eugenol could be dimerized in up to 80% yield to form the biphenyl derivative 46, using an inexpensive ammonium salt as a supporting electrolyte (Scheme 13). 46 was subsequently allylated or methylated to produce derivatives 47. Epoxidation of the double bonds completed the sequence, affording two different monomers 48 for use in polymer synthesis. Both derivatives were subsequently incorporated into durable epoxy resins, which displayed flame-retarding properties.

**Scheme 14:**
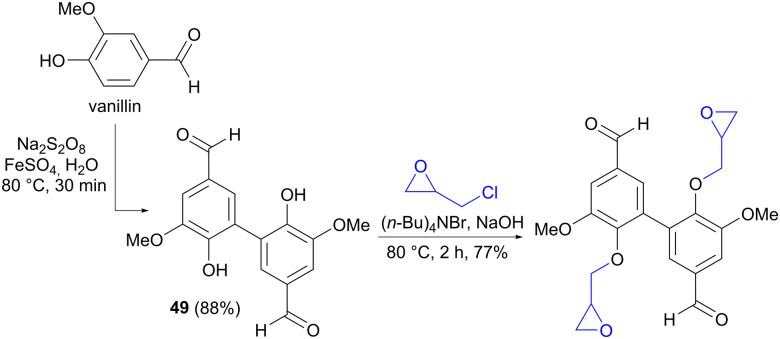
Oxidative coupling of vanillin to divanillin 49 using an oxidant.

It is worth mentioning that similar oxidative couplings of phenols can also be performed using an oxidizing reagent rather than by electrochemical means, as exemplified by the conversion of vanillin to divanillin (49) in the synthesis of a building block for epoxy resins (Scheme 14).^72^

**Scheme 15:**
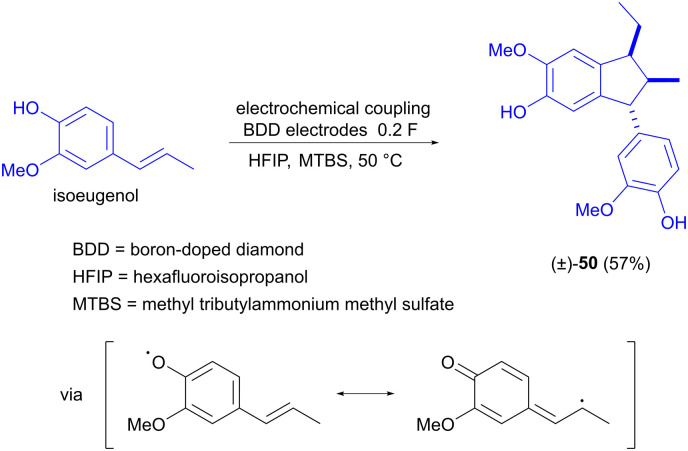
Stereoselective electrochemical coupling of isoeugenol.

The electrochemical dimerization of isoeugenol has also been investigated by Waldvogel and Einaga, but the product in this case is α-diisoeugenol (50, Scheme 15).^73^ The reaction is selective for stereoisomer 50, which is explained by the confinement of the two reacting molecules in a solvate cage, inducing a specific orientation that gives rise to the product stereoisomer shown. The difference in the coupling behaviour of eugenol compared to isoeugenol is due to the fact that the double bond of isoeugenol is conjugated with the aromatic ring. This allows the side chain to participate in the resonance stabilization of the initially formed oxygen radical and subsequently also in the carbon–carbon coupling reaction.

**Scheme 16:**
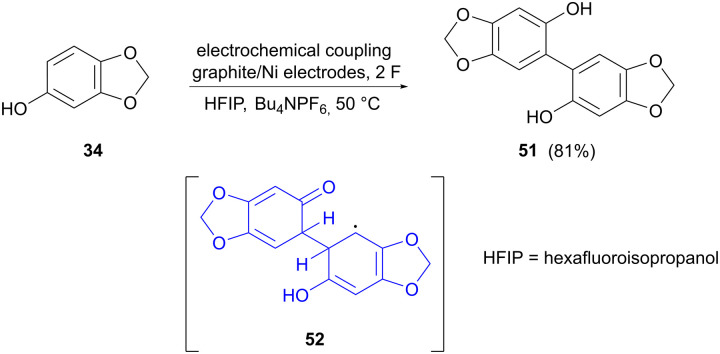
Electrochemical dimerization of sesamol, showing a potential intermediate in the reaction.

The last example of electrocatalytic phenolic dimerization concerns the coupling of sesamol (34) to form dimer 51, reported by Weidinger and colleagues (Scheme 16).^74^ The aim in this case was to provide more insight into the mechanistic aspects of electrooxidative coupling reactions. A suitable tool for this was found to be *in operando* Raman spectroscopy, which allows the observation of steady-state equilibria of reaction intermediates. Raman spectra were acquired at different stages of the reaction, showing new bands that could not be assigned to sesamol or the dimerized product. To probe the identity of these unknown bands, simulated Raman spectra for potential intermediates were compared to the actual recorded spectra, showing that radical dimer adduct 52 could be a possible intermediate in the reaction. The reaction behaviour in different solvents was also studied, showing that dimerization in methanol, as compared to hexafluoroisopropanol (HFIP), most likely proceeds *via* different reaction mechanisms, since MeOH can act as a redox mediator, which is not the case for HFIP with a higher redox potential.

## 7. Photocatalytic reactions

**Scheme 17:**
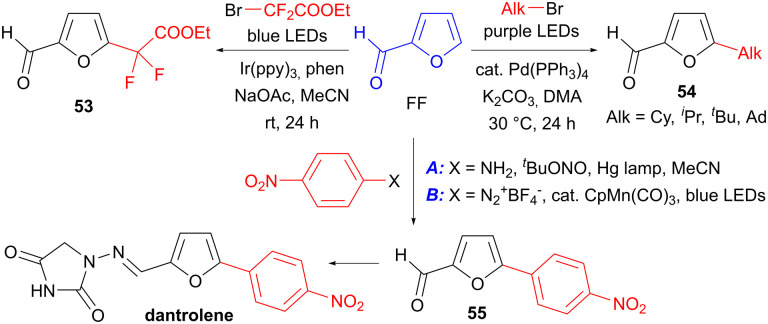
Functionalisation of FF using radical-mediated photochemical processes.

Light-induced transformations of bioderived arenes have been most extensively studied for bond cleavage reactions, *e.g.* in the depolymerisation of lignin.^75,76^ In contrast, C–C bond forming processes are comparatively underdeveloped, with most studies reporting methods for arene functionalisation which are applicable to a range of arenes (some of which may be bioderived) as opposed to studies focused on bioderived arenes specifically. Nevertheless, the ability of FF and other furanics to act as radical acceptors has allowed them to be derivatised in photochemical processes that use a variety of radical precursors. Thus, Li *et al.* were able to functionalise FF at the 5-position in a photoredox process using a difluoro-α-acyl radical to give 53 (Scheme 17).^77^ Alternatively, alkyl substituents may be introduced at the same position using the Pd-catalysed photochemical process reported by Chernyshev *et al.*, which gives products of type 54.^78^ The possibility of introducing aryl substituents is exemplified by the synthesis of *p*-nitrophenyl FF derivative 55, either using the corresponding aniline for the formation of a diazo anhydride intermediate from which the aryl radical is formed (conditions A),^79^ or using the corresponding diazonium salt in a manganese-catalysed photoredox process (conditions B).^80^ FF derivative 55 may be elaborated in one step to the muscle relaxant drug dantrolene.

**Scheme 18:**
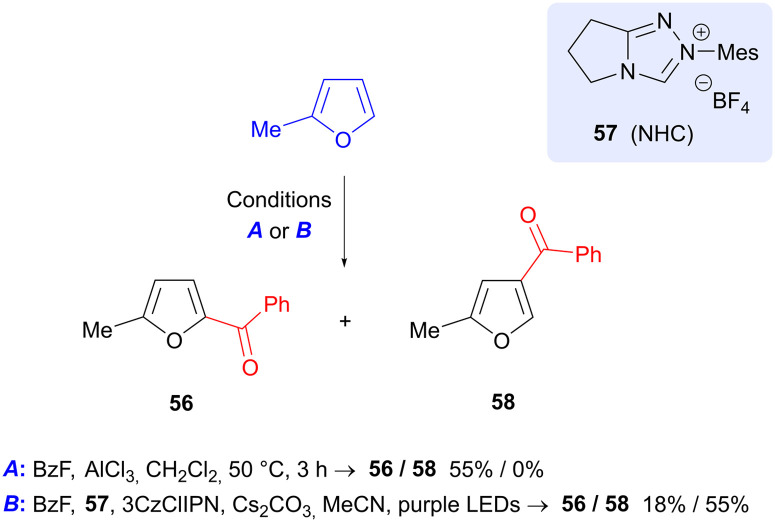
Regioselective benzoylation of 2-methylfuran *via* Lewis-acid catalyzed and photochemical processes.

2-Methylfuran (produced by reduction of FF) may be benzoylated in a process reported by Studer *et al.* using benzoyl fluoride, in which the regioisomer of product formed is dependent on the reaction conditions (Scheme 18).^81^ Use of aluminium trichloride gave 5-benzoyl product 56, in keeping with the expected S_E_Ar reactivity of furan. On the other hand, a photochemical process that employs 3CzClIPN as photocatalyst, in conjunction with N-heterocyclic carbene precursor 57, gave predominantly the 4-benzoyl isomer 58. Formation of 58 is proposed to occur by formation of a furan radical cation and an NHC-bound ketyl radical and their union.

## 8. Conclusions & outlook

The transition from using petroleum-based to bio-based precursors requires additional research to develop alternative synthetic routes to new and existing materials, fuels and pharmaceuticals. Nevertheless, moving towards more sustainable starting materials offers several advantages in this context. Firstly, functional groups that can be exploited for chemical transformations are already present in the molecules and do not have to be introduced in an extra step. In addition, lignin-based aromatics are adorned with alkyl groups and oxygenated substituents (hydroxy, methoxy), making them more electron-rich, and thus well suited for oxidative coupling reactions, as well as nucleophilic substitutions. We envisage many new exciting additions to the toolbox of C–C bond forming reactions using bio-based aromatics in the future.

## Author contributions

A. P. and P. D. Writing – original draft, review & editing; S. E. L. and N. K. Conceptualization, writing – original draft, review & editing.

## Data availability

No primary research results, software or code have been included and no new data were generated or analysed as part of this review.

## Conflicts of interest

There are no conflicts to declare.
